# Plastid genome variation in the green algal genus *Coelastrum* (Scenedesmaceae)

**DOI:** 10.3389/fpls.2026.1736783

**Published:** 2026-02-09

**Authors:** Chanhee Lee, Robert K. Jansen, Edward C. Theriot

**Affiliations:** 1 Plant Biology Graduate Program, University of Texas at Austin, Austin, TX, United States; 2 Department of Integrative Biology, University of Texas at Austin, Austin, TX, United States

**Keywords:** *Coelastrum*, intron distribution, phylogenomics, plastome evolution, plastome size variation, repetitive DNA

## Abstract

Plastid genomes (plastomes) in green algae display remarkable variation in size and structure, yet comprehensive species and strain-level analyses remain rare. Here, we present a detailed plastome comparison across 29 strains of nine nominal species of the genus *Coelastrum* (Scenedesmaceae). Sizes ranged from 166,827 bp to 553,457 bp, the latter representing the largest plastomes reported to date in the order Sphaeropleales. An almost twofold size difference was observed between strains of the same species, *Coelastrum morus*, highlighting unprecedented intraspecific plastome expansion in closely related green algae. Comparative analyses revealed that plastome size variation is primarily driven by the expansion of non-coding regions and repeats accumulation, with additional contributions from inverted repeat (IR) length and intron contents. Phylogenomic inference based on shared protein-coding genes recovered well-supported clades and resolved species-level relationships, offering improved taxonomic resolution relative to previous analyses based on several single gene analyses (nuclear ITS, nuclear SSU, *tufA*) which provided different relationships among critical taxa in *Coelastrum* and *Hariotina*. However, uneven taxon and strain sampling among molecular phylogenetic studies of *Coelastrum* and closely related Scenedesmaceae, including ours, is possibly as much of an obstacle to resolution of incongruences as is gene sampling. While gene content was largely conserved, we documented several lineage-specific gene and tRNA losses and unique intron insertions, reflecting dynamic structural evolution. Our results provide new insights into plastome architecture, intron evolution, and species boundaries within *Coelastrum*, and demonstrate the value of dense taxon and strain sampling for understanding plastid genome evolution in Chlorophyta.

## 1 Introduction

The plastid genome (plastome) of green algae typically exhibits a conserved quadripartite structure, consisting of two copies of an inverted repeat (IR) that encode the ribosomal RNA (rRNA) operon, separated by small single-copy (SSC) and large single-copy (LSC) regions (Lang and Nedelcu, 2012; Mower and Vickrey, 2018). Although this organization is shared with most land plants, plastome size and structure in green algae are notably more variable, ranging from as small as 64 kb to over 520 kb, compared to the 120–160 kb range in most photosynthetic seed plants (Wicke et al., 2011; Jansen and Ruhlman, 2012). For example, the plastome of *Prasinophyceae* sp. is only 64 kb in length with highly contracted intergenic regions comprising 10% of the genome (Lemieux et al., 2014). In contrast, *Haematococcus lacustris* and *H. pluvialis* have the largest green algal genomes discovered to date at over 1.35 Mb (Bauman et al., 2018; Ren et al., 2021). The primary contributor to large genome size in these and other green algae with plastomes over 500 kb, such as *Floydiella terrestris* and *Volvox carteri* are large non-coding intergenic regions which can account for up to 80% of their total genome size (Brouard et al., 2010; Smith and Lee, 2010; Smith, 2018; Ren et al., 2021).

Inverted repeat regions are evolutionarily dynamic across higher-level taxonomic categories in the Chlorophyta. There are examples of expansion or contraction in families as diverse as Chlorellaceae, Pedinomonadaceae, Prasiolaceae, and Trebouxiaceae (Turmel et al., 2017). More rarely, independent losses have been reported for *F. terrestris* (Chlorophyceae), *Stigeoclonium helveticum* (Chlorophyceae), *Bryopsis plumosa* (Ulvophyceae), and several Trebouxiophyceae species (Bélanger et al., 2006; Brouard et al., 2010; Turmel et al., 2015, 2016). Despite these insights, comprehensive studies of plastome evolution at the intraspecific or intrageneric level remain rare, limiting our understanding of structural diversity at lower taxonomic levels.

Plastome size variation in green algae is driven by several evolutionary factors, including differences in intron content, intergenic region size, IR variation, repeats, and gene loss. Intron distribution has been documented across major chlorophyte classes, including Trebouxiophyceae, Ulvophyceae, and Chlorophyceae (de Cambiaire et al., 2006, 2007; McManus et al., 2018; Turmel and Lemieux, 2018; Wang et al., 2019, 2024; Zhao et al., 2022). These studies span multiple plastid genes, such as *atpB*, *psaA*, *psaB*, *psbC*, *psbD*, and *rbcL*. The number, size, and lineage-specific presence of these introns have been shown to contribute significantly to plastome size and complexity (Muñoz-Gómez et al., 2017; McManus et al., 2018). Additionally, gene loss has been a pervasive force shaping plastome architecture in green algae. The ancestral green algal plastome is estimated to have encoded approximately 141 genes (Turmel and Lemieux, 2018) but substantial gene loss occurred across major lineages such as Prasinophytes and core Chlorophytes, including *Coelastrum*. Many of these losses have been attributed to gene transfers from the plastome to the nuclear genome (Stegemann et al., 2003; Palenik et al., 2007; Robbens et al., 2007; Turmel et al., 2009; Turmel and Lemieux, 2018). In Sphaeropleales, multiple genes involved in photosynthesis, plastid maintenance, and gene expression have been lost, reflecting a dynamic pattern of genome streamlining and functional reorganization (Fučíková et al., 2016).

Despite growing interest in plastome phylogenomics within green algae, detailed investigations of intron variation and gene loss at the species level remain limited, particularly within genera that include multiple closely related taxa. Most comparative analyses have focused on broad phylogenetic scales, leaving gaps in our understanding of how plastome evolution operates within specific clades (Lemieux et al., 2014; Turmel et al., 2015; Fučíková et al., 2016).

The genus *Coelastrum* (family Scenedesmaceae, order Sphaeropleales) represents an ideal group for addressing these gaps. Though *Coelastrum* is cosmopolitan and ecologically important, its organellar genome evolution remains largely unexplored. A molecular phylogenetic analysis by Hegewald et al. (2010) based on nuclear 18S ribosomal DNA (rDNA) and internal transcribed spacer 2 (ITS2) secondary structure suggested that *Coelastrum* may be non-monophyletic, with weakly supported interspecific relationships and several sister species relationships unresolved. In contrast, plastid genes such as *rbcL* and *tufA* have shown stronger phylogenetic signal in Scenedesmaceae and other chlorophytes (Sciuto et al., 2015), highlighting the need for plastome-level data to clarify relationships.

To date, only 19 complete plastomes have been published from the Scenedesmaceae (de Cambiaire et al., 2006; Wang et al., 2019, 2024, 2025; Douchi et al., 2021; Zhao et al., 2022; Cho and Lee, 2024; Xu et al., 2024), of which only one was a *Coelastrum* (Lee et al., 2023). In this paper, we added 28 newly sequenced plastomes from different strains of *Coelastrum*, for a total of 29 *Coelastrum* strains, spanning 9 nominal species, and compared them to 18 outgroup strains representing 18 species or subspecific taxa in 8 genera. We examined variation in genome size, IR boundaries, intron content, repeat element, and gene loss to provide an in-depth genomic perspective on organellar evolution within *Coelastrum*.

## 2 Materials and methods

### 2.1 Taxon sampling and DNA extraction

We reconstructed the plastome of 28 strains of *Coelastrum*. We isolated 11 new strains of *Coelastrum* from Gull Lake (3 strains), Swan Lake (4 strains), and Wintergreen Lake (2 strains) in Michigan, Coral Gables Canal in Florida (1 strain), and Lake Buchanan (1 strain) in Texas, all collected using a 20 μm mesh plankton net. The isolated strains were cultured and maintained in WC (Wright Chu) artificial freshwater media (Guillard, 1975). Another eight strains were obtained from the UTEX Culture Collection, and ten strains were acquired from the Experimental Phycology and Culture Collection of Algae (EPSAG). Additionally, one *Coelastrum* plastome and 18 Scenedesmaceae outgroup plastomes were downloaded from NCBI (http://www.ncbi.nlm.nih.gov/) for phylogenetic analysis: *Coelastrum microporum* (NC068582), *Asterarcys* sp. (MK995333), *Coccoidesmus tetrasporum* (OR350844), *Coelastrella saipanensis* (NC042181), *Coronastrum ellipsoideum* (PP979513), *Crucigenia lauterbornii* (PP979532), *Crucigenia quadrata* (PQ301446), *Desmodesmus abundans* (NC066651), *Desmodesmus* sp*inosus* (PV295633), *Pectinodesmus pectinatus* (NC036668), *Tetradesmus arenicola* (NC086756), *Tetradesmus bajacalifornicus* (NC086755), *Tetradesmus dimorphus* (NC086754), *Tetradesmus distendus* (NC086753), *Tetradesmus lancea* (OR502671), *Tetradesmus major* f. *lunatus* (OR502665), *Tetradesmus obliquus* (NC008101), *Tetradesmus obliquus* var. sp*iraliformis* (OR502672), and *Tetradesmus reginae* (NC086752).

All *Coelastrum* strains grown in our laboratory were observed under a light microscope for initial species identification based on morphological criteria, while cells were in exponential growth phase in WC media. Increases in cell abundance were determined by daily fluorescence in 25 mm diameter glass tubes in a Turner TD-700^®^ fluorometer. Scanning electron microscopy (SEM) was then utilized to evaluate ultrastructural variation in cell walls among species. Strains were fixed with formaldehyde or glutaraldehyde and dehydrated on a 25 mm diameter, 0.2 μm pore size membrane filter. Critical-point dried samples were mounted onto aluminum SEM stubs and coated with iridium using a sputter coater. Cell shape and ultrastructure were examined to confirm identity at the species levels based on authoritative references (Fenwick et al., 1966; Fenwick, 1968; Komárek and Fott, 1983; Hegewald et al., 2010).

Our identification of publicly available strains matched the names associated with those deposits and the literature. Our identification of our new strains was based upon agreement with the literature and with the morphology of the publicly available strains. We use the term “nominal species” to emphasize that any classification at any time is a hypothesis.

To obtain DNA, cultures grown in WC medium were harvested in exponential phase, and cells were pelleted by centrifugation at 4,500 rpm for 20 min. DNA was extracted from the collected pellets for next-generation sequencing (NGS) using a DNeasy^®^ Plant Mini Kit (Qiagen, Hilden, Germany) following the manufacturers protocol. DNA quantity was measured using the Qubit^®^ double stranded DNA High Sensitivity Assay Kit and the Qubit^®^ 2.0 Fluorometer, while DNA quality was assessed with the NanoDrop^®^ ND-1000 UV-Vis Spectrophotometer.

### 2.2 Plastome sequencing, assembly, and annotation

Total genomic DNA extracted from cultured *Coelastrum* strains was submitted to one of two facilities for high-throughput sequencing: the Genome Sequencing and Analysis Facility (GSAF) at the University of Texas at Austin or to Novogene (Beijing, China). For most strains, short-read sequencing libraries were prepared and sequenced on the Illumina HiSeq 4000 platform (Illumina, San Diego, CA), generating approximately 30 million paired-end reads (150 bp read length) per sample.

Short-read Illumina sequencing failed to yield complete plastome assemblies for three strains—SAG 2078, SAG 2248, and SAG 41.86, thus, long-read sequencing was performed on these three strains using the PacBio Sequel II platform with library preparation and sequencing carried out by Novogene.

Adapter sequences and low-quality bases were removed using BBDuk from the BBTools software suite (https://jgi.doe.gov/data-and-tools/bbtools/). Clean short reads were assembled *de novo* using NOVOPlasty v4.2.1 (Dierckxsens et al., 2017) on the Texas Advanced Computing Center (TACC) supercomputing platform, using an optimized k-mer size of 33 and an insert size of 300 bp. Long-read data were assembled with ptGAUL (plastid Genome Assembly Using Long reads) (Zhou et al., 2023), which is specifically designed for accurate reconstruction of plastid genomes from long-read datasets.

All resulting plastome assemblies were imported into Geneious Prime v2020.2.4 (Biomatters Ltd., Auckland, New Zealand). Assembly completeness, gene order, and IR boundaries for newly sequenced strains were examined, and read mapping was performed using BBMap (Bushnell, 2014) to verify coverage uniformity and support assembly accuracy. Taxonomic verification was conducted using BLAST searches against the NCBI nucleotide database (Altschul et al., 1990) to detect possible contaminants and confirm species identity. Plastome sequences were annotated in Geneious based on homologous genes from closely related Scenedesmaceae taxa and further validated with tRNAscan-SE v2.0 (Lowe and Chan, 2016) for tRNA identification and RNAmmer v1.2 (Lagesen et al., 2007) for rRNA gene prediction.

IR boundaries and annotations for all strains not sequenced by our laboratory followed annotations downloaded from NCBI.

### 2.3 Phylogenetic analysis

Phylogenetic relationships were inferred using protein coding genes (CDSs) shared across all 29 *Coelastrum* plastomes and at least 16 of the 18 outgroup species (**Supplementary Table S3**). Coding sequences for these 62 genes were extracted from the annotated plastomes in Geneious. CDS sequences were aligned using MAFFT v. 7.450 (Katoh and Standley, 2013) with a default setting in Geneious, and the resulting alignment was used for phylogenetic inference. Missing genes were treated as missing data, and maximum likelihood phylogenies were constructed using IQ-TREE2 v1.6.12 (Minh et al., 2020) with 1,000 bootstrap replicates. The best-fit substitution model, GTR+F+R5, was selected by ModelFinder (Kalyaanamoorthy et al., 2017). The resulting phylogenetic tree was visualized in FigTree v1.4.3 (http://tree.bio.ed.ac.uk/software/figtree/).

The aligned data matrix is available from the authors upon request.

IR boundaries determined by methods below for newly obtained sequences, and from NCBI annotations for downloaded plastomes, were mapped onto the best ML tree under parsimony.

### 2.4 Intron analyses

Intron presence and distribution were assessed across the newly assembled plastomes of *Coelastrum* strains and *Coelastrum microporum* (NC068582). Intron-containing genes were identified manually from the annotated plastomes using Geneious, and exon-intron boundaries were inferred by alignment with homologous plastid sequences from other *Coelastrum* species and from the related Scenedesmaceae genera *Coelastrella*, *Pectinodesmus*, *Scenedesmus*, and *Tetradesmus* (de Cambiaire et al., 2006; Wang et al., 2019; Zhao et al., 2022; Wang et al., 2024). To confirm intron identity and detect sequence conservation, all intron sequences were queried against the NCBI nucleotide database using BLAST. For each plastome, the total number of introns and the cumulative intron length (bp) were recorded. Intron variation was tabulated across strains and visualized alongside a maximum likelihood phylogeny to assess patterns of intron gain and loss across *Coelastrum* strains.

To evaluate the relationship between intron variation and overall plastome size, Pearson correlation tests were performed using R v4.3.0. Specifically, correlations were assessed between total intron number and size, and non-coding region size. This analysis aimed to identify which genomic features contribute most significantly to plastome size variation within *Coelastrum*.

### 2.5 Repeat content analysis

Repeat content was analyzed across 29 *Coelastrum* plastomes to evaluate the contribution of repetitive DNA to genome size variation. Prior to analysis, one copy of the inverted repeat (IRA) was removed from each plastome to prevent redundancy. Tandem repeats were detected using Tandem Repeats Finder v.4.09 (Benson, 1999) via the web interface, with default parameters. The total number and cumulative length of tandem repeats were calculated for each plastome.

Dispersed repeats were detected by running a BLASTN search of each plastome against itself using BLAST v2.16.0+ (Altschul et al., 1990), with word size of 16 and a minimum identity threshold of 80%, following the methods of Lee et al. (2020). Dispersed repeats were identified by retaining BLAST hits whose aligned query and subject regions occurred at distinct, non-overlapping positions within each plastome. Self-hits were removed, and matches representing overlapping or adjacent tandem duplications were excluded to ensure that only dispersed repeats were retained. Total repeats and the proportion of repeat content were calculated for each plastome.

## 3 Results

### 3.1 Phylogenetic relationships

Our analysis of plastome data recovered monophyly of all strains of *Coelastrum* with 100% BS support (**Supplementary Figure S1**). Two nominal species were represented by only one strain each: *C. cambricum* (UTEX 2446), and *C. indicum* (SAG 2363). They were recovered along with *C. microporum* (UTEX 281) in a clade with 100% BS support. The latter species was represented by a total of seven strains. The other six were recovered in two separate clades, with strains UTEX 1354, CG1, CW5, and CS5 in one clade and strains CF1 and SAG 2292 in another. In short, plastome data suggested that the morphology associated with *C. microporum* may consist of several independent lineages. A clade comprising *C.* sp*haericum* (SAG 1.82 and SAG 32.81) and *C. proboscideum* (UTEX 184 and UTEX 282) had 100% BS support, but neither nominal species was resolved as monophyletic within this clade.

In contrast, all other nominal species were recovered as monophyletic with high BS support. All *C. reticulatum* strains and all *C. morus* strains were each resolved with 100% BS support and those clades were resolved as sister to one another with 100% BS support. All six *C. astroideum* strains (CW1, CG6, CS1, CS3, CS9, and SAG 33.88) and all four *C. pseudomicroporum* strains (CLB, SAG 2077, UTEX 1353, and UTEX 280) were each recovered as monophyletic with 100% BS support.

### 3.2 Plastome general features

The newly assembled plastome sequences had a mean coverage ranging from 563.1 to 2,847.8X for 150 bp pair-end Illumina reads, and from 56.3 to 116.0X for long-read PacBio sequencing (**Table 1**). All plastomes were fully annotated and deposited in the NCBI GenBank database, with accession numbers provided in **Table 1**. Each plastome displayed a typical quadripartite structure, consisting of a large single copy (LSC) region and a small single copy (SSC) region with two inverted repeats (IRA and IRB).

**Table 1:** Summary of plastome features across *Coelastrum* species.

Species		Strain name	GenBank	Average depth of coverage	Size (bp)							GC content (%)
					Plastome	LSC	SSC	IR	Intron	Coding region	Non-coding	
*Coelastrum*	*astroideum*	CG6	PX512535	1456.6	186,201	82,667	70,148	16,693	13,128	93,889	92,312	31.0
	*astroideum*	CS1	PX512536	771.0	178,665	75,932	65,987	18,373	15,133	93,698	84,967	30.1
	*astroideum*	CS3	PX512537	1605.2	175,331	72,367	66,700	18,132	8,744	94,540	80,791	30.3
	*astroideum*	CS9	PX512538	563.1	175,332	72,367	66,701	18,132	8,725	94,521	80,811	30.3
	*astroideum*	CW1	PX512539	1059.8	186,257	82,662	70,147	16,724	13,126	93,862	92,395	31.0
	*astroideum*	SAG33.88	PX512540	1472.3	180,843	76,458	66,863	18,761	13,478	94,527	86,316	30.4
	*cambricum*	UTEX2446	PX512541	1955.4	171,270	85,045	68,701	8,762	15,872	93,108	78,162	30.6
	*microporum*	CF1	PX512542	1632.0	166,932	83,536	65,694	8,851	14,692	91,083	75,849	30.8
	***microporum***	CG1	NC068582	612.2	169,961	85,914	66,611	8,718	15,400	92,014	77,947	31.2
	*microporum*	CS5	PX512543	1802.8	168,159	80,353	65,796	11,005	14,523	91,165	76,994	30.9
	*microporum*	CW5	PX512544	1828.7	168,103	80,344	65,783	10,988	14,524	91,165	76,938	30.9
	*microporum*	SAG2292	PX512545	1917.7	171,509	85,345	66,488	9,838	20,746	91,053	80,456	31.1
	*microporum*	UTEX281	PX512546	1363.4	186,093	98,646	72,147	7,650	11,169	93,335	92,758	30.9
	*microporum*	UTEX1354	PX512547	1571.6	170,796	80,475	65,797	12,262	17,216	91,165	79,631	31.0
	*morus*	SAG2078	PX512671	116.0	298,895	139,858	87,785	35,626	25,873	94,381	204,514	27.4
	*morus*	SAG2248	PX512672	102.5	552,967	223,506	199,160	65,144	67,640	98,170	454,797	30.5
	*morus*	SAG41.86	PX512673	56.3	553,457	224,087	199,103	65,144	67,640	98,170	455,287	30.5
	*proboscideum* var. *dilatatum*	UTEX282	PX512548	1783.9	203,945	103,673	80,474	9,899	18,490	93,866	110,079	30.1
	*proboscideum* var. *gracile*	UTEX184	PX512549	1365.4	208,184	103,669	79,203	12,656	18,487	92,993	115,191	30.3
	*pseudomicroporum*	CLB	PX512550	1957.2	179,623	89,320	68,767	10,768	29,032	91,022	88,601	32.1
	*pseudomicroporum*	SAG2077	PX512551	2847.8	167,559	84,152	65,347	9,030	16,714	91,021	76,538	31.4
	*pseudomicroporum*	UTEX280	PX512552	2189.6	167,578	84,254	65,204	9,060	16,647	91,090	76,488	31.4
	*pseudomicroporum*	UTEX1353	PX512553	1345.4	166,827	82,056	64,851	9,960	16,222	91,082	75,745	31.9
	*pseudomicroporum*	SAG2363	PX512554	1993.9	198,867	90,540	69,579	19,374	28,366	95,196	103,671	30.5
	*reticulatum*	CG10	PX512555	664.8	200,459	107,222	74,859	9,189	9,580	93,038	107,421	29.3
	*reticulatum*	SAG8.81	PX512556	1410.4	193,431	95,451	73,942	12,019	16,782	93,651	99,780	29.1
	*reticulatum*	UTEX1365	PX512557	1035.7	242,214	92,255	58,315	45,822	16,288	89,638	152,576	30.3
	*sphaericum*	SAG1.82	PX512558	1035.9	208,400	103,667	79,319	12,707	18,488	93,122	115,278	30.3
	*sphaericum*	SAG32.81	PX512559	1124.2	208,308	103,672	79,186	12,725	18,489	93,122	115,186	30.3

Genomes obtained from NCBI are indicated by species names in bold.

**Figure 1:**
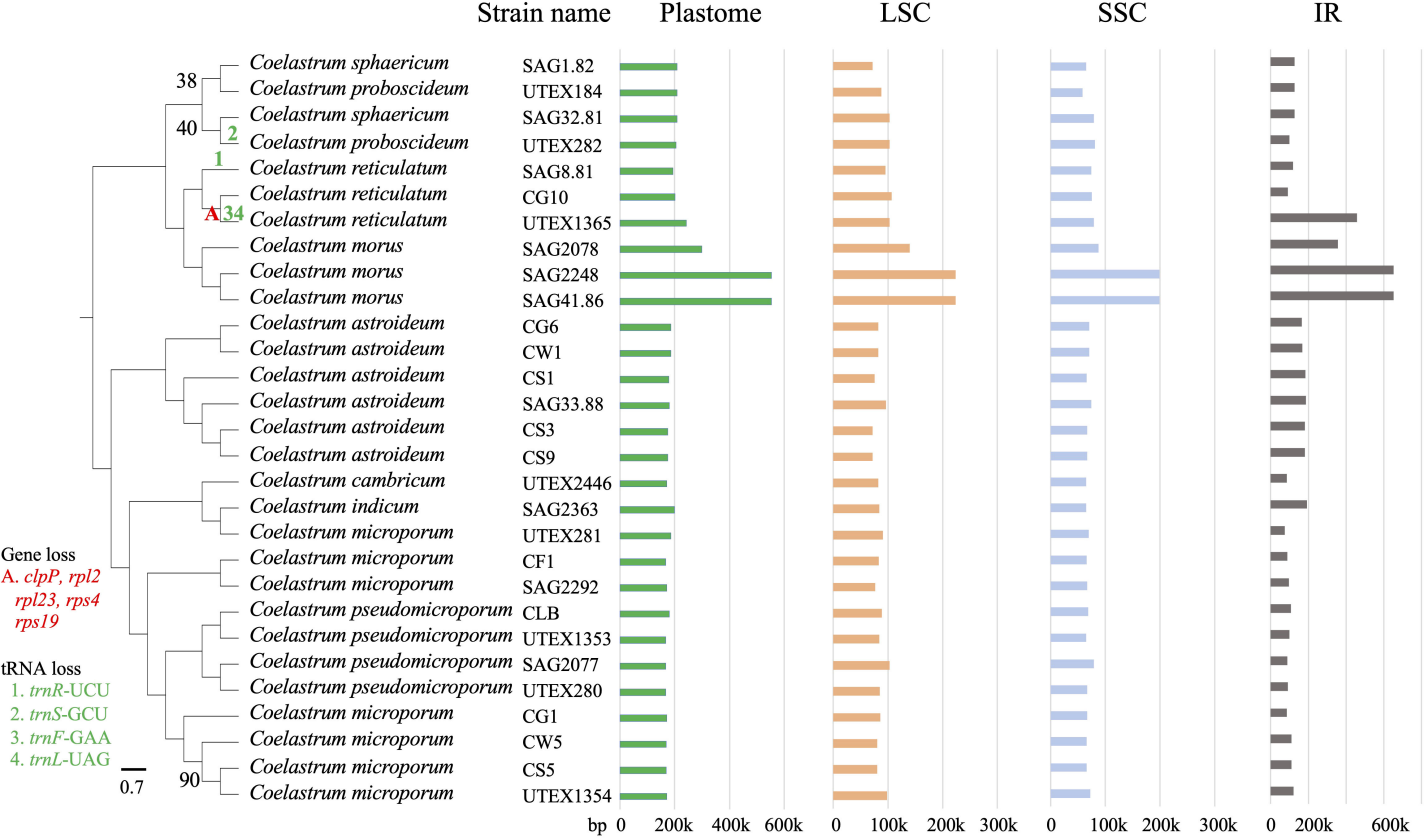
Phylogenetic relationships and plastome structural variation among 29 *Coelastrum* strains. The cladogram is based on the phylogram shown in **Supplementary Figure S1**. Bootstrap values less than 100% are indicated at the nodes. Variety names (*Coelastrum proboscideum* var. *gracile* UTEX184; *C. proboscideum* var. *dilatatum* UTEX282) were omitted from the figure labels for conciseness. Colored markers denote gene and tRNA losses mapped onto the relevant branches. Plastome, LSC, SSC and IR variation are indicated in bp (see also **Table 1**).

Despite this conserved structural organization, plastome sizes showed substantial interspecific variation, ranging from 166,827 bp in *Coelastrum pseudomicroporum* (UTEX 1353) to 553,457 bp in *Coelastrum morus* (SAG 41.86) (**Figure 1** and **Table 1**). The size of the single copy regions ranged from 137,407 bp to 423,190 bp (approximately a threefold range), whereas IR size ranged from 7,650bp to 65,144 bp, representing more than an eightfold range. These regions corresponded to 8.2% and 37.84% of total plastome size, respectively.

**Figure 2:**
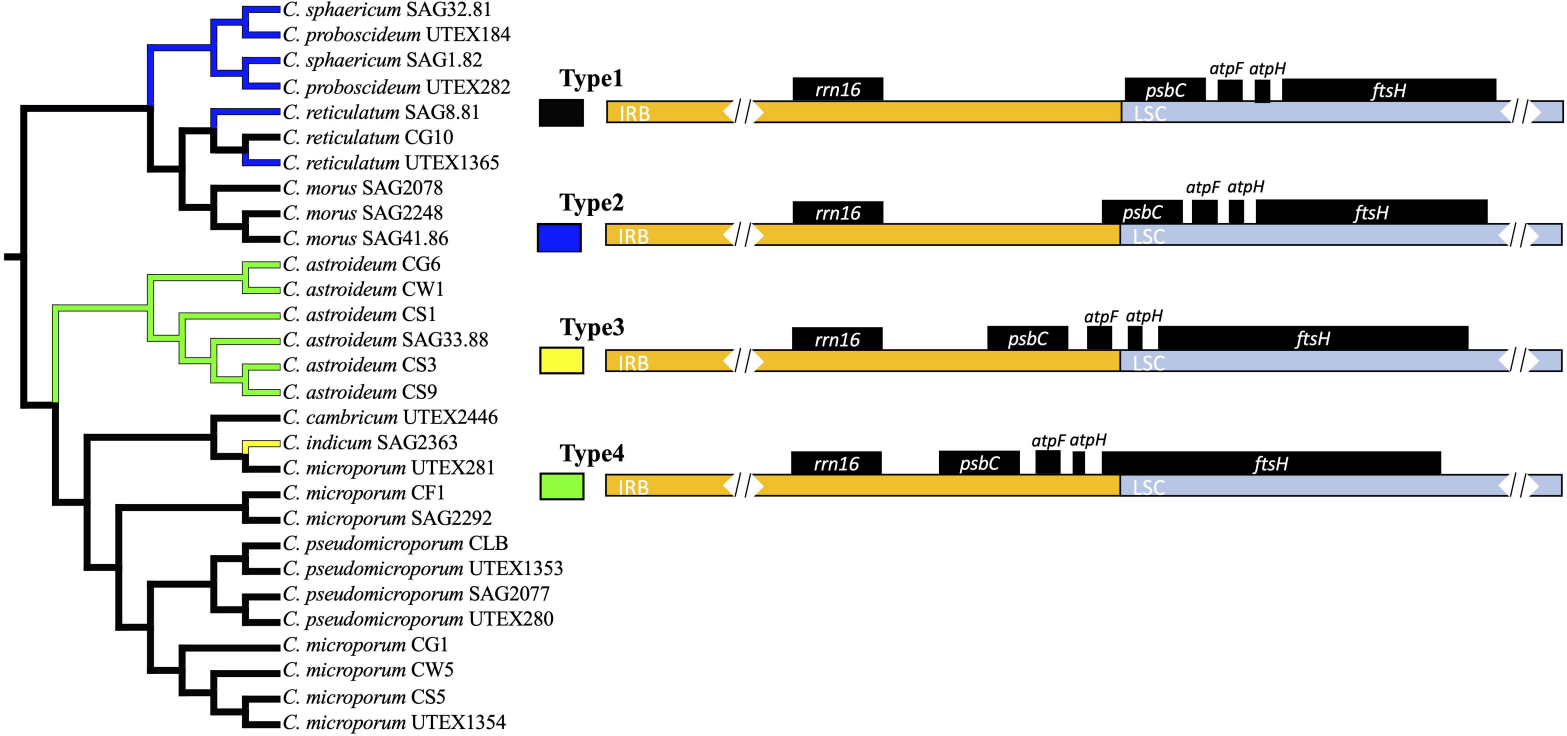
Inverted repeat (IR) boundary variation across major *Coelastrum* clades. Left: Phylogenetic tree of 29 *Coelastrum* strains and three outgroups, with branch colors indicating distinct IR structures. Right: Representative plastome maps showing IRB–LSC boundaries, with gene positions and duplications linked to IR expansion. Colors match the clades in the tree.

There were 4 types of inverted repeat boundaries (**Figure 2**). Inverted repeat expansion in certain lineages resulted in the translocation of four genes (*psbC*, *atpF*, *atpH*, and *ftsH*) across the LSC and the IR region. In Type 1, *psbC* was positioned in the LSC region near the IRB-LSC junction in the *C. morus* clade and in *C. reticulatum* CG10. It was also found in *C. cambricum* and all *C. microporum* and *C. pseudomicroporum*. The Type 2 boundary is similar, with the IR boundary bisecting the *psbC* gene, and was found in two *C. reticulatum* strains (SAG 8.81 and UTEX 1365) and in the *C.* sp*haericum* - C*. proboscideum* clade. Type 3 was restricted to *C. indicum* which retained *psbC* and *atpF* genes in the LSC but *atpH* and *ftsH* genes were found in the IRB region. Type 4 had a partial duplication of the *ftsH* gene within the IRB region of *C. astroideum*. Outgroup plastomes nearly all had the Type 1 boundary. *Pectinodesmus pectinatus* (NC036668) and *Tetradesmus obliquus* (NC008101) had Type 2 boundaries. *Crucigenia lauterbornii* (PP979532) and *Coronastrum ellipsoideum* (PP979513) had very different IR boundaries than any of the other plastomes. Regardless of where one might draw the root among outgroups, Type 1 still maps unambiguously as plesiomorphic to all of *Coelastrum*.

The total coding region length ranged from 89,638 bp to 98,170 bp (**Table 1**). Overall GC content across the genus was relatively consistent, ranging from 29.1% to 32.1%. However, one *C. morus* strain showed a lower GC content of 27.4%. In contrast, non-coding regions varied across strains, from 75,745 bp to 455,287 bp, comprising up to 82.3% of the total plastome length. This variation in non-coding content likely plays a central role in the plastome size differences observed among *Coelastrum* strains. To investigate the potential origins of the expanded intergenic regions in *C. morus*, we performed BLAST searches using representative intergenic sequences. These searches yielded no significant matches.

### 3.3 Gene loss and intron variation

Comparative analyses of gene content across 29 *Coelastrum* plastomes revealed overall conservation in core gene composition, with the exception of minor variation in tRNA gene content (**Supplementary Table S1**). The most pronounced case of gene loss was observed in *Coelastrum reticulatum* (UTEX 1365), which lacked five contiguous genes, *clpP*, *rpl2*, *rpl23*, *rps4*, and *rps19*, that are typically co-localized within the small single copy (SSC) region in other *Coelastrum* strains (**Figure 1**). This does not appear to be an artefact as coverage was deep and uniform across the region of interest (**Supplementary Figure S3**). In addition, *trnF*-GAA and *trnL*-TAG were also absent in this strain, suggesting either a localized deletion event or significant genomic rearrangement in the SSC region. Independent tRNA gene losses were detected in two additional strains: *trnR*-UCU was absent in *C. reticulatum* (SAG 8.81), and *trnS*-GCU was missing from *C. proboscideum* (UTEX 282).

**Figure 3:**
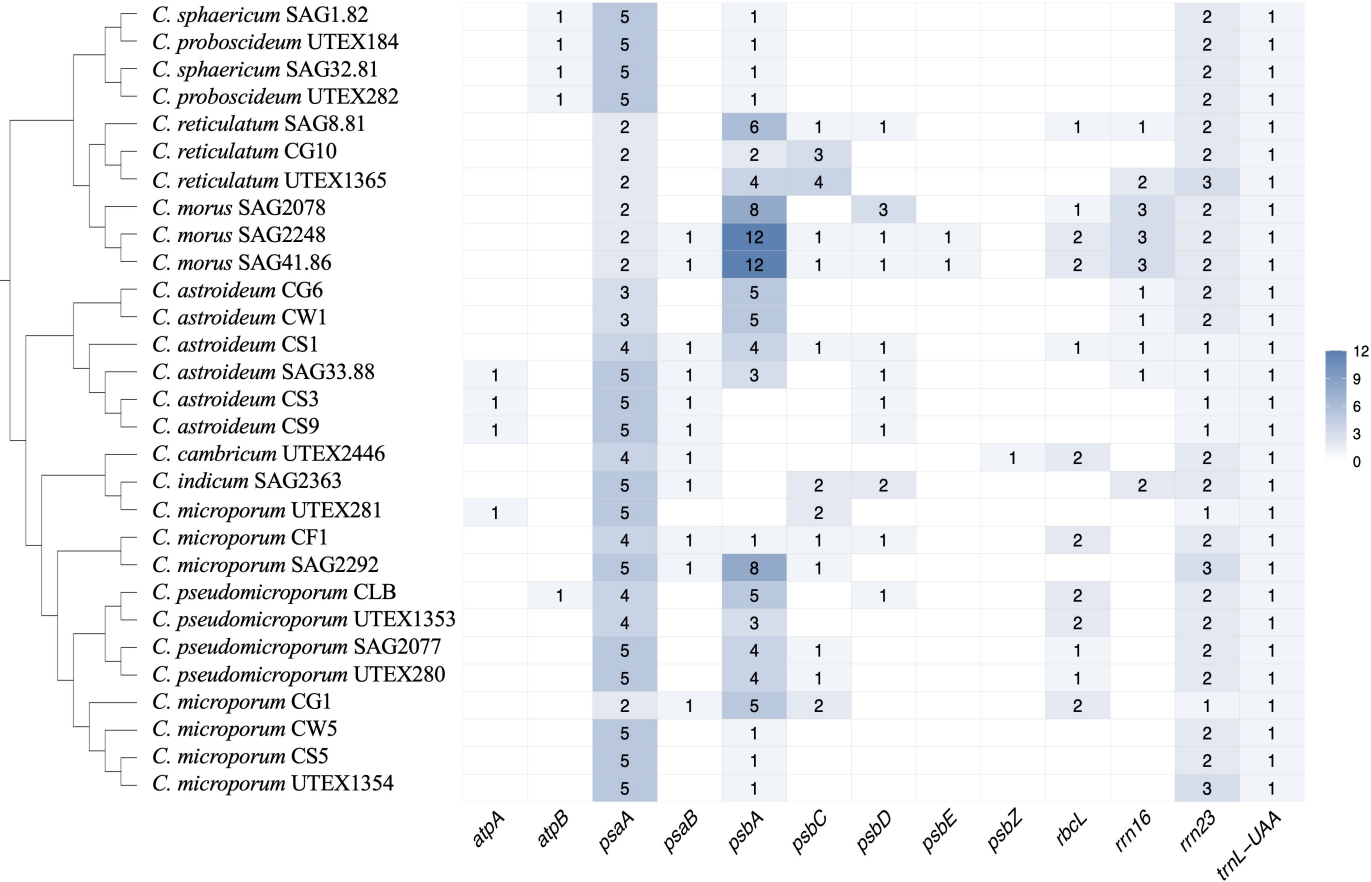
Heatmap of the number of introns across plastid genes for *Coelastrum* species. Numbers within cells denote the number of introns in each gene, while blank cells indicate the absence of introns.

Across the 29 assembled *Coelastrum* plastomes, intron distribution showed considerable heterogeneity in both location and frequency, reflecting dynamic evolutionary events. A total of 13 genes were identified as intron-bearing, with *psaA*, *psbA*, *psbC*, *rbcL*, and *rrn23* exhibiting the greatest variability in intron content and length (**Figure 3**). The *psaA* gene consistently displayed a trans-spliced structure with introns separating three exons. The cis-spliced configuration was observed in most *Coelastrum* strains.

Unique intron insertions were identified in several genes across the *Coelastrum* plastomes. Notably, an intron at position 66 in the *psbE* gene was detected in two of the three *C*. *morus* strains, but was absent in the third strain, indicating strain-specific intron retention or loss within the species. The *psbZ* intron (position 65) was observed only in *C. cambricum*, suggesting this to be a unique insertion event within *Coelastrum*. The *atpA* intron, located at site 489, was present in three strains of *C. astroideum* that did not form monophyletic group and one strain of *C. microporum* (UTEX 2446) in an unrelated clade. The canonical group I intron within *trnL*-UAA was conserved across all sampled plastomes, consistent with its widely reported vertical inheritance and evolutionary stability within Chlorophyta.

### 3.4 Plastome size variation

Four strains were outliers in terms of overall plastome size (**Table 1**). The two largest plastomes belonged to two *C. morus* strains (SAG 2248 and SAG 41.86). They were nearly identical in size to each other but were 85% larger than the third *C. morus* strain SAG 2078, which was 23% larger than that of *C. reticulatum* UTEX 1365. The latter was 16% larger than the next largest. After that, there was never more than a 4% difference in plastome size between any two strains. The reporting that follows refers to these four largest strains as the outliers, or as the four large strains.

**Figure 4:**
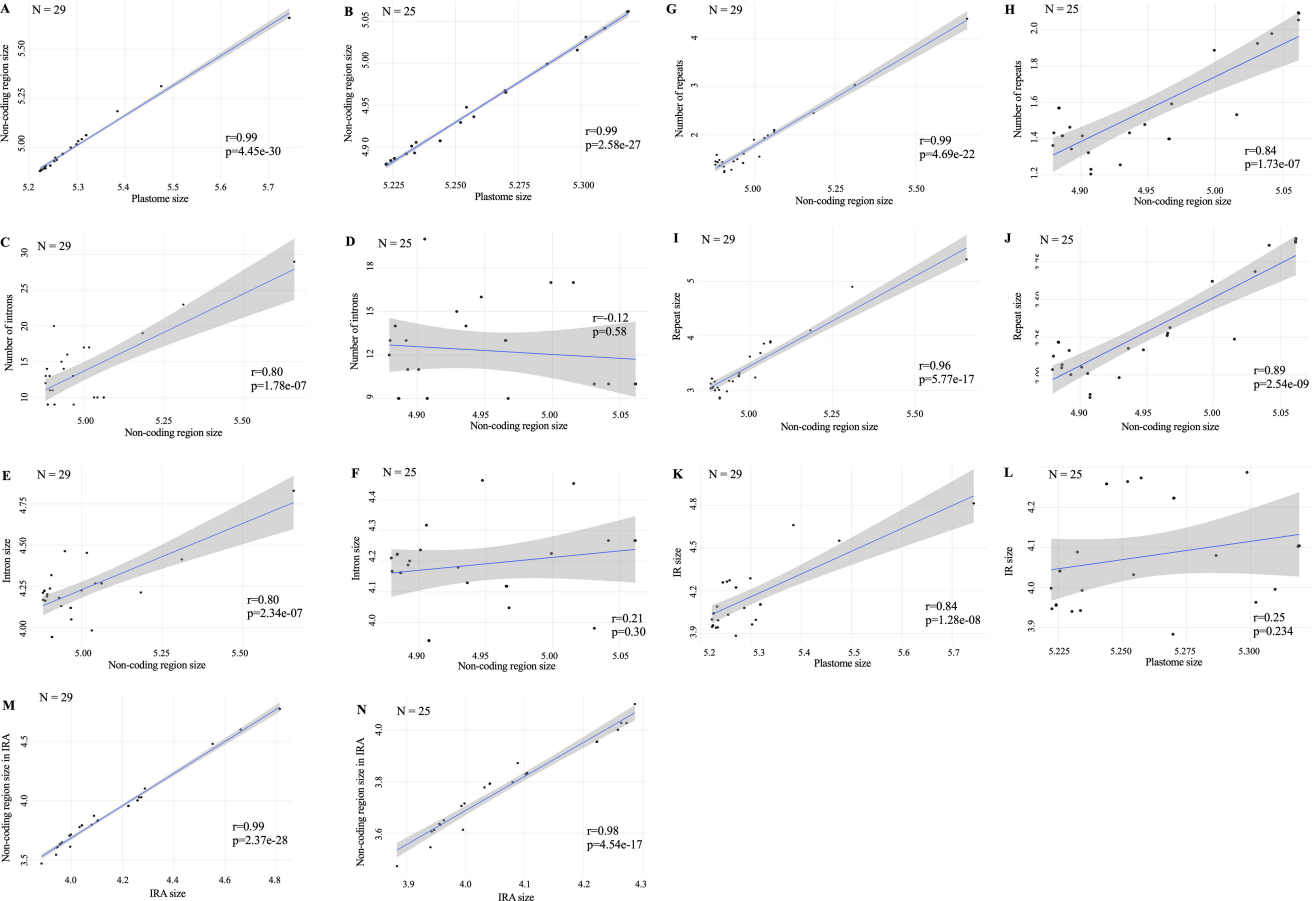
Pearson correlation between plastome size and key genomic features in *Coelastrum* strains with and without outliers defined in the text as *C. morus* strains SAG 2078, SAG 2248, and SAG 41.86, and *C. reticulatum* UTEX 1365 (N = 29; N = 25 respectively). Blue lines represent linear regression fits with 95% confidence intervals in gray shading. Pearson correlation coefficients (r) and p-values are shown in each graph. **(A, B)** Aggregate non-coding region size versus plastome size; **(C, D)** Number of introns versus aggregate non-coding region size; **(E, F)** Intron size versus aggregate non-coding region size; **(G, H)** Number of repeats versus aggregate non-coding region size; **(I, J)** Repeat size versus aggregate non-coding region size; **(K, L)** IR size versus plastome size; **(M, N)** Non-coding region size in IRA versus total IRA size.

There was relatively little variation in coding region size whether or not one considered outliers. For example, the coefficient of variation of the aggregate size of coding regions (from both single copy regions plus one IR copy) was about 2.2% with outliers and 1.5% without outliers (**Table 1**). The largest absolute contributor to plastome size was the non-coding region size which accounted for over 99% of the variation in total genome size whether or not outlier strains were included (r = 0.99, p < 0.001; **Figures 4A, B**).

We then analyzed contribution of various elements to aggregate non-coding region size. With outlier strains included, intron number and aggregate intron size were each positively correlated to non-coding size (**Figures 4C, E**) but removal of the outliers left no significant correlation (**Figures 4D, F**). However, regardless of whether or not outliers were included, total number and aggregate size of repeated elements were correlated with aggregate non-coding region size (**Figures 4G-J**).

That repeat elements were important contributors to size variation in all strains warranted more detailed investigation of these elements. Across the 29 *Coelastrum* plastomes, both dispersed and tandem repeats exhibited lineage-specific variation (**Table 2**). Repeat content was typically limited to 1–5 dispersed repeats per plastome, typically totaling <1,000 bp and contributing <0.5% of the genome. Tandem repeats in these compact plastomes were likewise limited, generally numbering fewer than 40 and spanning <2,000 bp, corresponding to <1% of total plastome size. In sharp contrast, repeat proliferation was dramatic in the *C. morus* lineage. The two giant plastomes, SAG 2248 and SAG 41.86, contained more than 26,000 dispersed repeats, representing ~200 kb of sequence and accounting for ~41% of their genome size. These same strains also harbored exceptionally high levels of tandem repeats, each exceeding 400 elements and ~53,000 bp in total length (~10% of the genome). Altogether, repetitive DNA surpassed 250 kb in these plastomes, contributing more than 50% of total genome size, whereas all other *Coelastrum* plastomes contained <5% repetitive DNA. Intermediate cases, such as *C. reticulatum* (UTEX 1365) and *C. morus* (SAG 2078), exhibited moderate repeat proliferation (100–120 dispersed repeats; ~7.5–8 kb), suggesting that repeat accumulation may have occurred in a stepwise and lineage-specific manner. These results again highlight repetitive DNA, both dispersed and tandem repeats, as a major driver of the genome size variation observed within *Coelastrum*.

**Table 2:** Dispersed and tandem repeat contents across 29 *Coelastrum* plastomes.

Strain name	Plastome size (removed IRA)	Dispersed repeat			Tandem repeat			Total repeat		
		Number	Total length (bp)	Content (%)	Number	Total length (bp)	Content (%)	Number	Length (bp)	Content (%)
UTEX1353	156,867	1	200	0.1	22	886	0.6	23	1,086	0.7
CF1	158,081	1	154	0.1	26	1,178	0.7	27	1,332	0.8
SAG2077	158,529	1	159	0.1	36	1,485	0.9	37	1,644	1.0
UTEX280	158,518	1	159	0.1	36	1,493	0.9	37	1,652	1.0
CW5	157,115	1	148	0.1	25	968	0.6	26	1,116	0.7
CS5	157,154	1	148	0.1	25	1,020	0.6	26	1,168	0.7
CG1	161,243	1	191	0.1	28	1,262	0.8	29	1,453	0.9
UTEX1354	158,534	1	148	0.1	25	979	0.6	26	1,127	0.7
UTEX2446	162,508	1	171	0.1	21	834	0.5	22	1,005	0.6
SAG2292	161,671	1	236	0.1	20	788	0.5	21	1,024	0.6
CS3	157,199	1	154	0.1	15	555	0.4	16	709	0.5
CS9	157,200	1	154	0.1	16	589	0.4	17	743	0.5
CS1	160,292	2	279	0.2	16	682	0.4	18	961	0.6
CLB	168,855	1	200	0.1	29	1,267	0.8	30	1,467	0.9
SAG33.88	162,082	5	578	0.4	22	923	0.6	27	1,501	0.9
UTEX281	178,443	1	146	0.1	38	1,916	1.1	39	2,062	1.2
CG6	169,508	5	716	0.4	20	1,110	0.7	25	1,826	1.1
CW1	169,533	5	716	0.4	20	1,177	0.7	25	1,893	1.1
SAG8.81	181,412	8	840	0.5	69	3,337	1.8	77	4,177	2.3
SAG2363	179,421	2	274	0.2	32	1,454	0.8	34	1,728	1.0
CG10	191,270	9	1,737	0.9	75	3,110	1.6	84	4,847	2.5
UTEX282	194,046	49	3,766	1.9	46	3,471	1.8	95	7,237	3.7
UTEX184	195,528	53	4,309	2.2	60	3,267	1.7	113	7,576	3.9
SAG32.81	195,583	65	4,446	2.3	59	3,280	1.7	124	7,726	4.0
SAG1.82	195,693	62	4,533	2.3	61	3,481	1.8	123	8,014	4.1
UTEX1365	196,392	179	7,627	3.9	100	5,043	2.6	279	12,670	6.5
SAG2078	263,269	975	71,475	27.1	121	8,151	3.1	1,096	79,626	30.2
SAG2248	487,823	26,446	200,674	41.1	420	53,018	10.9	26,866	253,692	52.0
SAG41.86	488,313	26,436	201,245	41.2	421	53,322	10.9	26,857	254,567	52.1

Samples are arranged in order of increasing plastome size. Total length of dispersed repeats is the total number of base pair sites occupied by all dispersed repeats.

The four outliers in overall plastome size also had the four largest IR sizes (**Table 1**). Size of the IR was positively correlated with total plastome size when outliers were included (**Figure 4K**), but not when outliers were excluded (**Figure 4L**). As for the entire plastome, non-coding DNA in the IR was positively correlated with IR size whether or not outliers were included (**Figures 4M, N**).

## 4 Discussion

Comparative analyses of complete plastomes for 29 *Coelastrum* strains provide insight into their evolution. The plastomes exhibit a typical quadripartite structure, but vary in total size, non-coding region content, and IR length. This strain-level plastome dataset suggests new insights into the structural, evolutionary, and phylogenetic diversity within *Coelastrum*.

We reiterate that we use the term “nominal species” to indicate that any classification is a hypothesis given the data at hand. Application of plastome data to Scenedesmaceae has thus far focused on higher level taxonomic relationships. Our application of plastome data to multiple strains of several *Coelastrum* nominal species speaks not only to higher level classification in the Scenedesmaceae but may also have revealed new evidence for cryptic species in *Coelastrum*.

### 4.1 Plastome size variation and causes of expansion

Our study reveals a nearly 3.3-fold variation in plastome size within *Coelastrum*, ranging from 166,827 bp in *C. pseudomicroporum* (UTEX 1353) to 553,457 bp in *C. morus* (SAG 41.86). A nearly two-fold difference in plastome size was observed between strains nominally identified as *C. morus*, representing an intraspecific size variation in green algae. The two strains of *C. morus* (SAG 2248 and SAG 41.86) stand out with an unprecedented genome size exceeding 550 kb, making them the largest plastomes reported within the order Sphaeropleales. Until now, plastomes of this magnitude have only been described in the green algal order Chlamydomonadales and Chaetopeltidales, such as *Volvox carteri*, *Floydiella terrestris*, *Haematococcus lacustris*, and *H. pluvialis* (Smith and Lee, 2009, 2010; Brouard et al., 2010; Bauman et al., 2018; Ren et al., 2021). These species often share several features including high GC content, repetitive DNA, and extensive intergenic regions. Plastome inflation has been proposed to be caused by error-prone DNA repair or mutational hazard in non-coding regions and repeat-rich regions (Smith and Lee, 2009; Brouard et al., 2010; Muñoz-Gómez et al., 2017; Smith, 2018, 2020; Ren et al., 2021).

In contrast, the plastomes of all four outliers, including the two largest *C. morus* strains, present a unique evolutionary case. Despite their extreme size, these four outliers do not exhibit unusually high GC content (range of GC = 27.4 - 30.5), nor do they show the remarkable intron density found in red algal lineages such as *Bulboplastis apyrenoidosa* and *Corynoplastis japonica*, which possess over 200 and 300 introns, respectively (Muñoz-Gómez et al., 2017). Instead, the two largest *C. morus* strains contain a modest 29 introns. Plastomes of the smaller outliers *C. morus* SAG 2078 and *C. reticulatum* UTEX 1365 contain only 23 and 19 introns. Taken together, these observations underscore that intron proliferation is not the main driver of non-coding region size and overall plastome enlargement in *Coelastrum*. This is particularly true when ignoring the outliers where we found lack of any significant correlation with intron number or size to total plastome size when outliers were removed from the analysis.

Our findings suggest that genome expansion in *Coelastrum* is primarily driven by the accumulation of non-coding sequence, particularly through the expansion of intergenic regions, rather than increased gene content or intron load. This mirrors patterns observed in *Haematococcus lacustris*, where plastome inflation is attributed to intergenic expansion (Smith, 2018), and in *B. apyrenoidosa*, where enlarged intergenic regions are linked to insertion sequences of bacterial origin (Muñoz-Gómez et al., 2017). Our BLAST search results of the expanded intergenic regions in *C. morus* yielded no significant matches, which may reflect limited genomic representation of closely related green algal taxa or potential bacterial donors in public databases.

In addition to intergenic expansion, repetitive DNA is a major driver of plastome enlargement. In the two largest *C. morus* plastomes, dispersed and tandem repeats exceed 250 kb, accounting for more than half of the total plastome length, whereas most *Coelastrum* plastomes contain only trace repeat content (<1%), with only a single dispersed repeat in approximately half of the samples. The magnitude of this proliferation parallels *Floydiella terrestris*, whose plastome is composed of nearly 50% short repeats (Brouard et al., 2010), and the repeat-rich architecture of *Haematococcus lacustris*, dominated by palindromic and dispersed repeats (Bauman et al., 2018; Smith, 2018). Within *Coelastrum*, repeat content scales with plastome size: compact genomes harbor little repetitive DNA, whereas enlarged lineages show progressively greater accumulations, culminating in the repeat-rich *C. morus* (**Table 2**). This pattern highlights repeat proliferation as a significant contributor to genome expansion. Although the exact molecular mechanisms remain elusive, inefficient DNA repair pathways, such as break-induced replication or the accumulation of palindromic repeats, may underlie intergenic expansion in *C. morus* plastomes, as proposed in other green and red algal systems (Muñoz-Gómez et al., 2017; Smith, 2020; Ren et al., 2021).

In summary, the pattern in *Coelastrum* aligns with previous findings in green algal lineages, where plastome enlargement has been attributed to the accumulation of non-coding DNA, repeats, IR, and introns (Brouard et al., 2010; Muñoz-Gómez et al., 2017; Turmel et al., 2017; Bauman et al., 2018; Cremen et al., 2018; Smith, 2018, 2020; Ren et al., 2021).

IR length ranged from 7,650 bp to 65,144 bp, contributing to differences in overall genome size. Multiple IR boundary shifts and gene duplications were observed, particularly involving *psbC*, *atpF*, *atpH*, and *ftsH*, indicating ongoing IR structural rearrangement. Patterns of IR boundary variation indicate that recently diverged lineages tend to exhibit more extensive IR expansions, reflected by the inclusion of additional genes into the IR (**Figure 2**). These findings align with the dynamic nature of IR expansion and contraction previously described in other members of green algae (Lemieux et al., 2014; Turmel et al., 2016).

*Tetradesmus* is the next most densely sampled Scenedesmaceae genus, with 13 strains (Cho and Lee, 2024). Total plastome size ranged from 148,816 to 196,309 bp. From this narrow perspective, the nearly tenfold range in total plastome size of *Coelastrum* is extraordinary, but we hope our findings will stimulate more detailed work on other Scenedesmaceae groups.

### 4.2 Intron dynamics

We focused our discussion on intron dynamics solely within *Coelastrum* because intron analysis has not been reported evenly across other Scenedesmaceae plastome studies. Nevertheless, Zhao et al. (2022) identified introns in seven single copy region genes, five of which also had introns in *Coelastrum*. The *rrn23* gene is the only *Pectinodesmus* IR gene with an intron; all *Coelastrum* strains have at least one intron in the same gene. *Coelastrella* has seven genes with introns, and all seven are shared with *Coelastrum* (Wang et al., 2019). *Coccoidesmus* was reported by Wang et al. (2024) to have introns in the large RNA subunit gene and in the *psbZ* gene. Introns were found in all of our large RNA subunit genes, but only in one *psbZ* gene (in our single strain of *C. cambricum* UTEX 2446, see **Supplementary Figure S2** and discussed below).

Intron diversity across the 29 *Coelastrum* plastomes revealed substantial lineage-specific variation in both presence and number, consistent with previous observations of dynamic intron evolution in Chlorophyta. Thirteen genes were identified as intron-bearing, with notable variability in *psaA*, *psbA*, *psbC*, *rbcL*, and *rrn23*. The distribution pattern largely mirrors those previously characterized in green algal plastomes, particularly in the order Sphaeropleales (Fučíková et al., 2016; McManus et al., 2018). The *psaA* gene consistently exhibited a trans-spliced architecture, with three exons interrupted by two introns, a synapomorphy of Sphaeropleales. This trans-splicing configuration is conserved across all *Coelastrum* strains sampled here. The cis-spliced configuration C1190 was absent in *C. microporum* (CG1) and the clade of *C. reticulatum* + *C. morus*. Within *Coelastrum*, this is most parsimoniously interpreted as parallel losses. The cis-spliced configuration is widely scattered across the Scenedesmaceae and certain *Pediastrum* species within the sister family Hydrodictyaceae (McManus et al., 2018), and we hesitate, without increased taxon sampling, to conclude whether this intron presence represents parallel gains across the Sphaeropleales, whether its absence represents parallel losses, or some more complex process.

With the caveat that taxon sampling and reporting of intron data are still uneven in the Scenedesmaceae, it does appear that several introns may be lineage-specific or represent recent insertion events. For instance, introns in *psbE* (position 66) and *psbZ* (position 65) were restricted to *C. morus* (SAG 2248 and SAG 41.86) and *C. cambricum* (UTEX 2446), respectively. As far as we are aware, there are no previous reports of introns in *psbE* in any green algae. Similarly, the *atpA* intron (position 489) was restricted to a clade within *C. astroideum* strains and to one *C. microporum* strain (UTEX 281). This intron is shared with the distantly related *Neochloris aquatica*, suggesting either deep homology or a horizontal transfer event (McManus et al., 2018). The group I intron in *trnL-UAA* was universally conserved across all *Coelastrum* plastomes. This intron has been reported as a vertically inherited feature across photosynthetic eukaryotes, from red algae to land plants (Besendahl et al., 2000), and its universal presence here further supports its evolutionary stability within the Chlorophyta.

A striking pattern of intron proliferation was observed in the *psbA* gene of *C. morus*. Strains SAG 2078, SAG 2248, and SAG 41.86 contained up to seven introns at unique insertion positions (81, 174, 273, 393, 486, 533, 741, 885), far exceeding the typical intron number (≤4) reported in *Scenedesmus obliquus* (de Cambiaire et al., 2006) and species within the sister family Hydrodictyaceae (McManus et al., 2018). This lineage-specific intron expansion suggests recent and rapid intron gain events.

### 4.3 Gene loss events

Most *Coelastrum* plastomes retain a conserved gene complement consistent with other Scenedesmaceae. However, *C. reticulatum* (UTEX 1365) exhibits a unique gene loss cluster affecting five adjacent protein-coding genes (*clpP*, *rpl2*, *rpl23*, *rps4*, *rps19*) within the SSC region, along with tRNA genes *trnF*-GAA and *trnL*-TAG. Similar clustered gene loss events have been reported in other green algae and are thought to reflect localized rearrangements, deletions, or functional transfers to the nucleus (Brouard et al., 2010; Turmel and Lemieux, 2018). Additional tRNA gene losses in *C. reticulatum* (SAG 8.81), *C. proboscideum* (UTEX 282), and outgroups such as *Pectinodesmus pectinatus* suggest that tRNA genes are particularly prone to loss or replacement. The gene *clpP* encodes a subunit of the chloroplast Clp protease, while *rpl2, rpl23, rps4*, and *rps19* encode ribosomal protein subunits, suggesting that the loss of these genes may affect plastid protease or ribosome function. Transcriptomic analysis/nuclear genome sequencing needs to be conducted to confirm loss or transfer of these genes to the nucleus.

### 4.4 Phylogenetic implications

Our recovery of *Coelastrum* as monophyletic highlights issues of both gene and taxon sampling in interpreting the phylogeny of the Scenedesmaceae. It is beyond the scope of this paper to resolve issues related to these problems, but we focus on two papers that clearly highlight them.

Hegewald et al. (2010) used a relatively short sequence (ITS2). Nevertheless, we consider this to be a fundamental study because of its breadth of taxon sampling, including 106 Scenedesmaceae strains from eleven nominal genera. In contrast to our results, they recovered a non-monophyletic *Coelastrum*. Two strains are of particular interest because we selected them for analysis because they occupied critical positions in the Hegewald et al. (2010) tree. Namely, our study and Hegewald et al. (2010) included SAG 2078 (*C. morus*) and SAG 8.81 (*H. reticulata* Dangeard = *C. reticulatum* (Dangeard) Senn). If one were to trim away strains unique to only one study or the other, *C. reticulatum* SAG 8.81 would be sister to *C. morus* SAG 2078 in both studies. However, differences in taxon sampling exist, and one cannot ignore that Hegewald et al. (2010) also recovered *Coelastrella* spec. SAG 217.5 (isolated from Finland), *Dimorphococcus lunatus* SAG 2241, and *Asterarcys quadricellulare* COMAS 1977–75 as close relatives to both SAG 8.81 and *Coelastrum cambricum* SAG 7.81. The latter strain was not available at the time we assembled cultures and data for analysis, so we used *C. cambricum* UTEX 2446. ITS2 data provided only weak support at the node linking *Coelastrella* spec. SAG 217.5 to other taxa, whereas plastome data using the already published *Coelastrella saipanensis* sequence (NC042181), placed that *Coelastrella* strain in a position distant from all three of our *C. reticulatum* strains, including SAG 8.81. It is possible that *Coelastrum saipanensis* (NC042181) and *C*. spec. SAG 217.5 belong to different lineages, and/or that other taxon sampling differences influenced the relationship of *Coelastrella* to *Coelastrum*. We also note that the deeper nodes of the *Coelastrum sensu lato* branch had very low BS support in Hegewald et al. (2010), indicating that differences between their tree and ours may also be due to gene sampling and low resolving power of the ITS2 at critical nodes.

A much more recent paper used multiple longer sequences in three independent analyses, and again illustrated that both gene and strain sampling affect tree topologies. Wang et al. (2024) analyzed nuclear ITS data (referred to only as the “ITS region” in that study), the nuclear SSU (18S), and the plastome gene *tufA* independently of one another, each with a similar but not identical taxon sampling design. *Coelastrum* was recovered as monophyletic with 18S data, but the only two species included were *C. proboscideum* (SAG 217-3) and *C.* sp*haericum* (SAG 217-2). *Hariotina reticulata* (SAG 8.81) was embedded in a monophyletic *Hariotina* and that genus was several nodes away from *Coelastrum*. However, neither genus was monophyletic when ITS data were analyzed, and different *Coelastrum* strains (*C. astroideum* var. *rugosum* RW10 and *C. microporum* FNY-1) were employed. Finally, with *tufA* data, and using a third pair of *Coelastrum* (*C.* sp*haericum* CCMA UFSCar 060, and *C.* sp. YN 15-2), a monophyletic *Coelastrum* was recovered as sister to a monophyletic *Hariotina*.

We are not criticizing Wang et al. (2024), as they used sequences available in NCBI, and they discussed several points of incongruence. We only use Hegewald et al. (2010) and Wang et al. (2024) to illustrate that molecular phylogenetic studies need to account for strain and taxon sampling as well as gene sampling when discussing classification. Finally, we note that the SSU and ITS data are from one compartment (nuclear genome) and the *tufA* data are from another (the plastome). The possibility of distinct gene trees and organismal trees must be considered as well when attempting to corroborate or reject monophyly for *Coelastrum*.

In summary, future studies of classification using phylogenomic data obviously need to include both more outgroup and ingroup taxa, and more strain representatives for each species, to conclusively support or reject *Coelastrum* as monophyletic.

Looking towards lower-level classifications, towards the tips of the plastome tree, there is general agreement between species names and plastome genetic diversity. Plastome data also recovered hierarchical structure within nominal species. Discovery of additional diversity within a historical morphological classification at the species level by the use of DNA sequence data often leads to the invocation of “cryptic species”, but morphology in algae is usually treated in some sort of non-canonical fashion, whereas molecular data are treated phylogenetically (Alverson, 2008).

The phylogenetic species concept demands only that a species be the smallest monophyletic group that a systematist cares to name. That is, grouping is objective, but naming is arbitrary (Frost and Kluge, 1994; Alverson, 2008; Mishler and Theriot, 2000). Multiple strains of each of *C. morus*, *C. reticulatum*, *C. astroideum*, and *C. pseudomicroporum* are each supported as monophyletic with BS support of 100%.

However, phylogenetic structure was recovered within each of these clades, with plastome size and sequence contributing to diversity in all four clades (**Figure 1** and **Table 1**). The clade of *C. morus* strains recovered two (SAG 2248 and SAG 41.86) with plastomes of over 500 kb and a third (SAG 2078) whose genome was almost 300 kb (compared to a median of about 180 kb). The two largest plastomes could represent one phylogenetic species and SAG 2078 could possibly represent a second. Similarly, there was one strain of *C. reticulatum* (UTEX 1365) with a plastome nearly 40 kb larger than that of *C. reticulatum* CG10, and nearly 48 kb larger than that of *C. reticulatum* SAG 8.81. *Coelastrum asteroideum* is represented by at least two distinct clades each receiving 100% BS support (strains CG6 and CW1; strains CS1, CS3, CS9, and SAG 33.88). The former two strains had nearly identical sequences, and also the largest plastomes among nominal *C. asteroideum* differing in length by only 56 bases between the two. However, the Swan Lake strains (CS1, CS3, and CS9) differed by about 3 kb, and SAG 33.88 was another 2 kb larger than the largest Swan Lake strain (6 kb smaller than the CG6 and CW1 strains). Plastomes of *C. pseudomicroporum* were recovered in two subclades (CLB and UTEX 1353; SAG 2077 and UTEX 280). Sister strains UTEX 1353 and CLB had the smallest and largest plastomes of *C. pseudomicroporum*, being nearly 13 kb difference in size.

Plastome size differences within these *Coelastrum* clades ranged from 100 bp differences to several hundred thousand bp differences. The biological meaning is unclear and there are no clear guidelines to be taken from the general literature on Viridiplantae plastome size and species diagnosis. For example, in vascular plants, species of pears are known to interbreed when plastome sizes differ in terms of only a few hundred bases (Kim et al., 2024). On the other hand, differences in plastome size of as much as 20 kb are considered intraspecific in the widely cultivated *Peucedanum japonicum* (Joh et al., 2025). We are confident that increasing strain sampling in green microalgae will find other such perplexing and interesting observations.

The non-monophyly of *C. microporum* strains presents a different set of issues. There are three distinct lineages. One strain (UTEX 281) is embedded within a clade that includes *C. indicum* (SAG 2363) and *C. cambricum* (UTEX 2446), while CF1 and SAG 2292 form a clade of strains with nearly identical sequences, as do CS5, CW5 and UTEX 1354. Again, we observe variation among nominal strains of *C. microporum* of as much as 20 kb. These inconsistencies may reflect simple taxonomic misidentification, incomplete lineage sorting, and/or historical hybridization events.

Two species mentioned several times in various studies are particularly in need of revision and probably need to be considered synonymous. *Coelastrum* sp*haericum* and *C. proboscideum* have been difficult to resolve both with morphology and genetic data. Komárek and Fott (1983) claimed that these species differ in apical structure, with the former being formed by small bumps, and the latter representing a crown-like structure. Others have considered the two species synonymous (Hajdu et al., 1976). Our results are consistent with the findings of Hegewald et al. (2010), who suggested a persistent phylogenetic signal despite differences in molecular markers used (e.g., nuclear ITS). Such a signal, interpreted as shared apomorphic similarity in morphology and DNA sequence, strongly supports the classification of *C.* sp*haericum* and *C. proboscideum* as a single phylogenetic species. We note again, however, that the largest plastome is more than 4 kb larger than the smallest. Thus, such inferences will be more powerful once broader strain sampling is done across both data sets.

In summary, phylogenetic resolution may be constrained by limited taxon sampling (Hillis, 1998; Pollock et al., 2002; Zwickl and Hillis, 2002; Heath et al., 2008), especially in cases where only a single strain represents a species. In such instances, intraspecific variability remains unassessed, which may mask deeper population structure or contribute to apparent paraphyly. Finding strains that are very similar in coding region sequence, with shared morphological characteristics, but very different plastome sizes, in all of our clades underscores the need to expand strain sampling across multiple populations per species to better understand species classification and diagnosis in these green algae.

## 5 Conclusion

This study presents the most comprehensive species and strain-level analysis of plastid genome evolution in *Coelastrum*, a morphologically diverse and ecologically important genus within the Scenedesmaceae. We demonstrate that plastome size variation in *Coelastrum* is primarily explained by the expansion of intergenic regions and accumulation of repetitive DNA, with additional contributions from variation in IR length and, in some cases, intron content. While most plastomes retain a conserved gene complement, lineage-specific gene loss and intron dynamics highlight the ongoing evolutionary plasticity of these organellar genomes. We observed an almost twofold difference in plastome size among strains of the same nominal species, *C. morus*. There was a less impressive but still large difference in plastome size among lineages in *C. reticulatum* (that of UTEX 1365 was 16% larger than the other two strains).

The plastome-based phylogeny reveals well-resolved relationships among species and supports the utility of plastome data for refining taxonomic classification in green algae. These findings contribute to our understanding of structural genome evolution in Chlorophyta and underscore the importance of expanding sampling at the species and strain levels in plastome studies.

Future work integrating nuclear and mitochondrial genomes, along with ecological data, will help resolve the evolutionary forces shaping organelle genome architecture in *Coelastrum* and other green algal lineages. Although classification was not the focus of our study, our plastome analysis also identified areas of congruence and incongruence between morphology and molecular markers, which bear further study.

## Data Availability

The datasets presented in this study can be found in online repositories. The names of the repository/repositories and accession number(s) can be found below: https://figshare.com/articles/dataset/Complete_annotated_chloroplast_genome_sequences_of_29_i_Coelastrum_i_strains_Sphaeropleales_Chlorophyta_/30482909
