# Supplementary material for: Plastid genome variation in the green algal genus Coelastrum (Scenedesmaceae)
Source: Front Plant Sci. 2026 Feb 9;17:1736783. doi: 10.3389/fpls.2026.1736783 (PMC12926389; doi:10.3389/fpls.2026.1736783)
Supplement: Supplementary file 1 [file DataSheet1.docx]

Supplementary Figure 1. Maximum likelihood phylogram of *Coelastrum* species and 18 outgroup taxa based on concatenated plastid protein-coding genes. Bootstrap values below 100% are shown at the nodes. Newly assembled plastomes are indicated by species and strain names in bold. The GenBank accession numbers corresponding to the Coelastrum strains are listed in Table 1.

Supplementary Figure 2. Distribution of introns in the plastomes of *Coelastrum* examined in this study. Numbers indicate insertion site within genes.

Supplementary Figure 3. Mapping results showing the average coverage depth of the plastome in *Coelastrum reticulatum* UTEX1365 to verify the presence of an essential gene loss event in the small single copy (SSC) region.


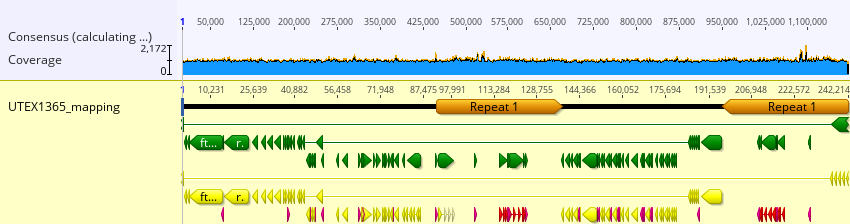


Supplementary Table 1. List of shared plastid genes in Coelastrum, organized by functional group. Genes highlighted with bold indicate the five genes that are absent in a single Coelastrum *reticulatum* strain, UTEX 1365, but present in all other strains analyzed.

| ATP synthase | *atpA, atpB, atpE, atpF, atpH, atpI* |
| --- | --- |
| Cytochrome b6f complex | *petA, petB, petD, petG, petL* |
| Photosystem I | *psaA, psaB, psaC, psaJ* |
| Photosystem II | *psbA, psbB, psbC, psbD, psbE, psbF, psbH, psbI, psbJ, psbK, psbL, psbM, psbN, psbT, psbZ* |
| RuBisCO large subunit | *rbcL* |
| RNA polymerase | *rpoA, rpoB, rpoC1, rpoC2* |
| Ribosomal proteins (SSU) | *rps2, rps3,* ***rps4****, rps7, rps8, rps9, rps11, rps12, rps14, rps18,* ***rps19*** |
| Ribosomal proteins (LSU) | ***rpl2****, rpl5, rpl12, rpl14, rpl16, rpl20,* ***rpl23****, rpl36* |
| Ribosomal RNAs | *rrn5, rrn16, rrn23* |
| Transfer RNAs | *trnA*-UGC, *trnC*-GCA, *trnD*-GUC, *trnE*-UUC, *trnF*-GAA, *trnG*-GCC, *trnG*-UCC, *trnH*-GUG, *trnI*-CAU, *trnI*-GAU, *trnK*-UUU, *trnL*-UAA, *trnL*-UAG, *trnM*-CAU, *trnM*-CAU, *trnN*-GUU, *trnP*-UGG, *trnQ*-UUG, *trnR*-ACG, *trnR*-UCU, *trnS*-UGA, *trnS*-GCU, *trnT*-UGU, *trnV*-UAC, *trnW*-CCA, *trnY*-GUA |
| Hypothetical chloroplast reading frames | *tufA, ycf1, ycf3, ycf4, ycf12* |
| Other genes | *ccsA, cemA, chlB, chlL, chlN,* ***clpP****, ftsH, infA* |

| Strain name | Coding size in IR | Non-coding size in IR | Intron size in IR | Intron number in IR | Tandem repeat number in IR | Tandem repeat sizein IR | The number of | | | | | | | Intergenic region GC (%) | Gene density |
| --- | --- | --- | --- | --- | --- | --- | --- | --- | --- | --- | --- | --- | --- | --- | --- |
|  |  |  |  |  |  |  | Total genes | Unique genes | Unique CDS | Introns | tRNA | Unique tRNA | rRNA |  |  |
| CG6 | 7662 | 9,031 | 2890 | 3 | 7 | 540 | 107 | 97 | 68(3) | 13 | 30(4) | 26(3) | 3(3) | 28.2 | 0.57 |
| CS1 | 7664 | 10,709 | 2824 | 3 | 1 | 58 | 107 | 97 | 68(3) | 15 | 30(4) | 26(3) | 3(3) | 26.8 | 0.60 |
| CS3 | 8056 | 10,076 | 1015 | 1 | 4 | 176 | 107 | 97 | 68(3) | 9 | 30(4) | 26(3) | 3(3) | 27.0 | 0.61 |
| CS9 | 8056 | 10,076 | 1015 | 1 | 4 | 176 | 107 | 97 | 68(3) | 9 | 30(4) | 26(3) | 3(3) | 27.0. | 0.61 |
| CW1 | 7662 | 9,062 | 2890 | 3 | 7 | 607 | 107 | 97 | 68(3) | 13 | 30(4) | 26(3) | 3(3) | 28.2 | 0.57 |
| SAG33.88 | 8056 | 10,705 | 1314 | 2 | 4 | 251 | 107 | 97 | 68(3) | 14 | 30(4) | 26(3) | 3(3) | 27.4 | 0.59 |
| UTEX2446 | 4725 | 4,037 | 1914 | 2 | 0 | 0 | 104 | 97 | 68 | 11 | 30(4) | 26(3) | 3(3) | 27.9 | 0.61 |
| CF1 | 4761 | 4,090 | 2316 | 2 | 3 | 142 | 104 | 97 | 68 | 13 | 30(4) | 26(3) | 3(3) | 27.2 | 0.62 |
| CG1 | 5216 | 3,502 | 1416 | 1 | 7 | 441 | 104 | 97 | 68 | 13 | 30(4) | 26(3) | 3(3) | 28.0 | 0.61 |
| CS5 | 4793 | 6,212 | 4255 | 2 | 1 | 86 | 104 | 97 | 68 | 9 | 30(4) | 26(3) | 3(3) | 27.3 | 0.62 |
| CW5 | 4793 | 6,195 | 4255 | 2 | 2 | 90 | 104 | 97 | 68 | 9 | 30(4) | 26(3) | 3(3) | 27.3 | 0.62 |
| SAG2292 | 4761 | 5,077 | 3325 | 3 | 2 | 67 | 104 | 97 | 68 | 20 | 30(4) | 26(3) | 3(3) | 28.0 | 0.61 |
| UTEX281 | 4696 | 2,954 | 967 | 1 | 4 | 214 | 104 | 97 | 68 | 9 | 30(4) | 26(3) | 3(3) | 28.8 | 0.56 |
| UTEX1354 | 4793 | 7,469 | 5601 | 3 | 3 | 128 | 104 | 97 | 68 | 11 | 30(4) | 26(3) | 3(3) | 27.6 | 0.61 |
| SAG2078 | 5369 | 30,257 | 5054 | 5 | 21 | 1670 | 103 | 97 | 68 | 23 | 29(3) | 26(3) | 3(3) | 25.5 | 0.34 |
| SAG2248 | 5486 | 59,658 | 16581 | 5 | 63 | 5674 | 104 | 97 | 68 | 29 | 30(4) | 26(3) | 3(3) | 30.2 | 0.19 |
| SAG41.86 | 5486 | 59,658 | 16581 | 5 | 63 | 5674 | 104 | 97 | 68 | 29 | 30(4) | 26(3) | 3(3) | 30.2 | 0.19 |
| UTEX282 | 5800 | 4,099 | 2209 | 2 | 6 | 289 | 103 | 96 | 68 | 10 | 28(3) | 25(2) | 3(3) | 27.8 | 0.51 |
| UTEX184 | 5890 | 6,766 | 2209 | 2 | 21 | 1357 | 104 | 97 | 68 | 10 | 30(4) | 26(3) | 3(3) | 28.5 | 0.50 |
| CLB | 4768 | 6,000 | 4245 | 2 | 1 | 34 | 104 | 97 | 68 | 16 | 30(4) | 26(3) | 3(3) | 29.7 | 0.58 |
| SAG2077 | 4707 | 4,323 | 2457 | 2 | 4 | 181 | 104 | 97 | 68 | 14 | 30(4) | 26(3) | 3(3) | 28.3 | 0.62 |
| UTEX280 | 4776 | 4,284 | 2388 | 2 | 4 | 189 | 104 | 97 | 68 | 14 | 30(4) | 26(3) | 3(3) | 28.3 | 0.62 |
| UTEX1353 | 4768 | 5,192 | 3415 | 2 | 1 | 34 | 104 | 97 | 68 | 12 | 30(4) | 26(3) | 3(3) | 28.8 | 0.62 |
| SAG2363 | 6675 | 12,699 | 9646 | 6 | 3 | 261 | 106 | 97 | 68 | 17 | 30(4) | 26(3) | 3(3) | 28.6 | 0.53 |
| CG10 | 4725 | 4,464 | 1576 | 2 | 14 | 661 | 104 | 97 | 68 | 10 | 30(4) | 26(3) | 3(3) | 26.4 | 0.52 |
| SAG8.81 | 5740 | 6,279 | 3092 | 4 | 9 | 628 | 103 | 96 | 68 | 17 | 29(4) | 25(3) | 3(3) | 25.6 | 0.53 |
| UTEX1365 | 5858 | 39,964 | 8759 | 9 | 56 | 3169 | 101 | 92 | 63 | 19 | 31(7) | 24(5) | 3(3) | 28.8 | 0.42 |
| SAG1.82 | 5890 | 6,817 | 2209 | 2 | 20 | 1392 | 104 | 97 | 68 | 10 | 30(4) | 26(3) | 3(3) | 28.5 | 0.50 |
| SAG32.81 | 5890 | 6,835 | 2209 | 2 | 20 | 1387 | 104 | 96 | 68 | 10 | 30(4) | 26(3) | 3(3) | 28.3 | 0.50 |

Supplementary Table 2. Summary of plastome features across Coelastrum species. This table provides extended information complementing the summary in Table 1. Numbers in parentheses indicate gene copies duplicated within the inverted repeat (IR) regions.

Supplementary Table 3. Presence–absence matrix of 62 protein-coding genes (CDSs) shared across all 29 Coelastrum plastomes and present in at least 16 of the 18 outgroup strains. Only genes missing in individual strains are indicated by “N”.

|  | CF1 | CG1 | CG10 | CG6 | CLB | CS1 | CS3 | CS5 | CS9 | CW1 | CW5 | SAG1.82 | SAG2077 | SAG2078 | SAG2248 | SAG2292 | SAG2363 | SAG32.81 | SAG33.88 | SAG41.86 | SAG8.81 | UTEX1353 | UTEX1354 | UTEX1365 | UTEX184 | UTEX2446 | UTEX280 | UTEX281 | UTEX282 | MK995333 | NC008101 | NC036668 | NC042181 | NC066651 | NC086752 | NC086753 | NC086754 | NC086755 | NC086756 | OR350844 | OR502665 | OR502667 | OR502671 | OR502672 | PP979532 | PQ301446 | PV295633 |
| --- | --- | --- | --- | --- | --- | --- | --- | --- | --- | --- | --- | --- | --- | --- | --- | --- | --- | --- | --- | --- | --- | --- | --- | --- | --- | --- | --- | --- | --- | --- | --- | --- | --- | --- | --- | --- | --- | --- | --- | --- | --- | --- | --- | --- | --- | --- | --- |
| *atpA* |  |  |  |  |  |  |  |  |  |  |  |  |  |  |  |  |  |  |  |  |  |  |  |  |  |  |  |  |  |  |  |  |  |  |  |  |  |  |  |  |  |  |  |  |  |  |  |
| *atpB* |  |  |  |  |  |  |  |  |  |  |  |  |  |  |  |  |  |  |  |  |  |  |  |  |  |  |  |  |  |  |  |  |  |  |  |  |  |  |  |  |  |  |  |  |  |  |  |
| *atpE* |  |  |  |  |  |  |  |  |  |  |  |  |  |  |  |  |  |  |  |  |  |  |  |  |  |  |  |  |  |  |  |  |  |  |  |  |  |  |  |  |  |  |  |  |  |  |  |
| *atpF* |  |  |  |  |  |  |  |  |  |  |  |  |  |  |  |  |  |  |  |  |  |  |  |  |  |  |  |  |  |  |  |  |  |  |  |  |  |  |  | N |  |  |  |  |  |  |  |
| *atpH* |  |  |  |  |  |  |  |  |  |  |  |  |  |  |  |  |  |  |  |  |  |  |  |  |  |  |  |  |  |  |  |  |  |  |  |  |  |  |  |  |  |  |  |  |  |  |  |
| *atpI* |  |  |  |  |  |  |  |  |  |  |  |  |  |  |  |  |  |  |  |  |  |  |  |  |  |  |  |  |  |  |  |  |  |  |  |  |  |  |  |  |  |  |  |  |  |  |  |
| *ccsA* |  |  |  |  |  |  |  |  |  |  |  |  |  |  |  |  |  |  |  |  |  |  |  |  |  |  |  |  |  |  |  |  |  |  |  |  |  |  |  |  |  |  |  |  |  |  |  |
| *cemA* |  |  |  |  |  |  |  |  |  |  |  |  |  |  |  |  |  |  |  |  |  |  |  |  |  |  |  |  |  |  |  |  |  |  |  |  |  |  |  |  |  |  |  |  |  |  |  |
| *chlB* |  |  |  |  |  |  |  |  |  |  |  |  |  |  |  |  |  |  |  |  |  |  |  |  |  |  |  |  |  |  |  |  |  |  |  |  |  |  |  |  |  |  |  |  |  |  |  |
| *chlL* |  |  |  |  |  |  |  |  |  |  |  |  |  |  |  |  |  |  |  |  |  |  |  |  |  |  |  |  |  |  |  |  |  |  |  |  |  |  |  |  |  |  |  |  |  |  |  |
| *chlN* |  |  |  |  |  |  |  |  |  |  |  |  |  |  |  |  |  |  |  |  |  |  |  |  |  |  |  |  |  |  |  |  |  |  |  |  |  |  |  |  |  |  |  |  |  |  |  |
| *ftsH* |  |  |  |  |  |  |  |  |  |  |  |  |  |  |  |  |  |  |  |  |  |  |  |  |  |  |  |  |  | N |  |  |  |  |  |  |  |  |  |  |  |  |  |  |  |  |  |
| *infA* |  |  |  |  |  |  |  |  |  |  |  |  |  |  |  |  |  |  |  |  |  |  |  |  |  |  |  |  |  |  |  |  |  |  |  |  |  |  |  |  |  |  |  |  |  |  |  |
| *petA* |  |  |  |  |  |  |  |  |  |  |  |  |  |  |  |  |  |  |  |  |  |  |  |  |  |  |  |  |  |  |  |  |  |  |  |  |  |  |  |  |  |  |  |  |  |  |  |
| *petB* |  |  |  |  |  |  |  |  |  |  |  |  |  |  |  |  |  |  |  |  |  |  |  |  |  |  |  |  |  |  |  |  |  |  |  |  |  |  |  |  |  |  |  |  |  |  |  |
| *petD* |  |  |  |  |  |  |  |  |  |  |  |  |  |  |  |  |  |  |  |  |  |  |  |  |  |  |  |  |  |  |  |  |  |  |  |  |  |  |  |  |  |  |  |  |  |  |  |
| *petG* |  |  |  |  |  |  |  |  |  |  |  |  |  |  |  |  |  |  |  |  |  |  |  |  |  |  |  |  |  |  |  |  |  |  |  |  |  |  |  |  |  |  |  |  |  |  |  |
| *petL* |  |  |  |  |  |  |  |  |  |  |  |  |  |  |  |  |  |  |  |  |  |  |  |  |  |  |  |  |  |  |  |  |  |  |  |  |  |  |  |  |  |  |  |  |  |  |  |
| *psaA* |  |  |  |  |  |  |  |  |  |  |  |  |  |  |  |  |  |  |  |  |  |  |  |  |  |  |  |  |  |  |  |  |  |  |  |  |  |  |  | N |  |  |  |  |  |  |  |
| *psaB* |  |  |  |  |  |  |  |  |  |  |  |  |  |  |  |  |  |  |  |  |  |  |  |  |  |  |  |  |  |  |  |  |  |  |  |  |  |  |  |  |  |  |  |  |  |  |  |
| *psaC* |  |  |  |  |  |  |  |  |  |  |  |  |  |  |  |  |  |  |  |  |  |  |  |  |  |  |  |  |  |  |  |  |  |  |  |  |  |  |  |  |  |  |  |  |  |  |  |
| *psaJ* |  |  |  |  |  |  |  |  |  |  |  |  |  |  |  |  |  |  |  |  |  |  |  |  |  |  |  |  |  |  |  |  |  |  |  |  |  |  |  |  |  |  |  |  |  |  |  |
| *psbA* |  |  |  |  |  |  |  |  |  |  |  |  |  |  |  |  |  |  |  |  |  |  |  |  |  |  |  |  |  | N |  |  |  |  |  |  |  |  |  |  |  |  |  |  |  | N |  |
| *psbB* |  |  |  |  |  |  |  |  |  |  |  |  |  |  |  |  |  |  |  |  |  |  |  |  |  |  |  |  |  |  |  |  |  |  |  |  |  |  |  |  |  |  |  |  |  |  |  |
| *psbC* |  |  |  |  |  |  |  |  |  |  |  |  |  |  |  |  |  |  |  |  |  |  |  |  |  |  |  |  |  |  |  |  |  |  |  |  |  |  |  |  |  |  |  |  |  |  |  |
| *psbD* |  |  |  |  |  |  |  |  |  |  |  |  |  |  |  |  |  |  |  |  |  |  |  |  |  |  |  |  |  |  |  |  |  |  |  |  |  |  |  |  |  |  |  |  |  |  |  |
| *psbE* |  |  |  |  |  |  |  |  |  |  |  |  |  |  |  |  |  |  |  |  |  |  |  |  |  |  |  |  |  |  |  |  |  |  |  |  |  |  |  |  |  |  |  |  |  |  |  |
| *psbF* |  |  |  |  |  |  |  |  |  |  |  |  |  |  |  |  |  |  |  |  |  |  |  |  |  |  |  |  |  | N |  |  |  |  |  |  |  |  |  |  |  |  |  |  |  |  |  |
| *psbH* |  |  |  |  |  |  |  |  |  |  |  |  |  |  |  |  |  |  |  |  |  |  |  |  |  |  |  |  |  |  |  |  |  |  |  |  |  |  |  |  |  |  |  |  |  |  |  |
| *psbI* |  |  |  |  |  |  |  |  |  |  |  |  |  |  |  |  |  |  |  |  |  |  |  |  |  |  |  |  |  |  |  |  |  |  |  |  |  |  |  |  |  |  |  |  |  |  |  |
| *psbJ* |  |  |  |  |  |  |  |  |  |  |  |  |  |  |  |  |  |  |  |  |  |  |  |  |  |  |  |  |  |  |  |  |  |  |  |  |  |  |  |  |  |  |  |  |  |  |  |
| *psbK* |  |  |  |  |  |  |  |  |  |  |  |  |  |  |  |  |  |  |  |  |  |  |  |  |  |  |  |  |  |  |  |  |  |  |  |  |  |  |  |  |  |  |  |  |  |  |  |
| *psbL* |  |  |  |  |  |  |  |  |  |  |  |  |  |  |  |  |  |  |  |  |  |  |  |  |  |  |  |  |  |  |  |  |  |  |  |  |  |  |  |  |  |  |  |  |  |  |  |
| *psbM* |  |  |  |  |  |  |  |  |  |  |  |  |  |  |  |  |  |  |  |  |  |  |  |  |  |  |  |  |  |  |  |  |  |  |  |  |  |  |  |  |  |  |  |  |  |  |  |
| *psbN* |  |  |  |  |  |  |  |  |  |  |  |  |  |  |  |  |  |  |  |  |  |  |  |  |  |  |  |  |  |  |  |  |  |  |  |  |  |  |  |  |  |  |  |  |  | N | N |
| *psbT* |  |  |  |  |  |  |  |  |  |  |  |  |  |  |  |  |  |  |  |  |  |  |  |  |  |  |  |  |  |  |  |  |  |  |  |  |  |  |  |  |  |  |  |  |  |  |  |
| *psbZ* |  |  |  |  |  |  |  |  |  |  |  |  |  |  |  |  |  |  |  |  |  |  |  |  |  |  |  |  |  |  |  |  |  |  |  |  |  |  |  |  |  |  |  |  |  |  |  |
| *rbcL* |  |  |  |  |  |  |  |  |  |  |  |  |  |  |  |  |  |  |  |  |  |  |  |  |  |  |  |  |  |  |  |  |  |  |  |  |  |  |  |  |  |  |  |  |  | N |  |
| *rpl5* |  |  |  |  |  |  |  |  |  |  |  |  |  |  |  |  |  |  |  |  |  |  |  |  |  |  |  |  |  |  |  |  |  |  |  |  |  |  |  |  |  |  |  |  |  |  |  |
| *rpl12* |  |  |  |  |  |  |  |  |  |  |  |  |  |  |  |  |  |  |  |  |  |  |  |  |  |  |  |  |  |  |  |  |  |  |  |  |  |  |  |  |  |  |  |  |  |  |  |
| *rpl14* |  |  |  |  |  |  |  |  |  |  |  |  |  |  |  |  |  |  |  |  |  |  |  |  |  |  |  |  |  |  |  |  |  |  |  |  |  |  |  |  |  |  |  |  |  |  |  |
| *rpl16* |  |  |  |  |  |  |  |  |  |  |  |  |  |  |  |  |  |  |  |  |  |  |  |  |  |  |  |  |  |  |  |  |  |  |  |  |  |  |  |  |  |  |  |  |  |  |  |
| *rpl20* |  |  |  |  |  |  |  |  |  |  |  |  |  |  |  |  |  |  |  |  |  |  |  |  |  |  |  |  |  |  |  |  |  |  |  |  |  |  |  | N |  |  |  |  |  | N |  |
| *rpl36* |  |  |  |  |  |  |  |  |  |  |  |  |  |  |  |  |  |  |  |  |  |  |  |  |  |  |  |  |  | N |  |  |  |  |  |  |  |  |  |  |  |  |  |  |  |  |  |
| *rpoA* |  |  |  |  |  |  |  |  |  |  |  |  |  |  |  |  |  |  |  |  |  |  |  |  |  |  |  |  |  |  |  |  |  |  |  |  |  |  |  |  |  |  |  |  |  |  |  |
| *rpoB* |  |  |  |  |  |  |  |  |  |  |  |  |  |  |  |  |  |  |  |  |  |  |  |  |  |  |  |  |  | N |  |  |  |  |  |  |  |  |  |  |  |  |  |  |  |  |  |
| *rpoC1* |  |  |  |  |  |  |  |  |  |  |  |  |  |  |  |  |  |  |  |  |  |  |  |  |  |  |  |  |  |  |  |  |  |  |  |  |  |  |  | N |  |  |  |  |  |  |  |
| *rpoC2* |  |  |  |  |  |  |  |  |  |  |  |  |  |  |  |  |  |  |  |  |  |  |  |  |  |  |  |  |  |  |  |  |  |  |  |  |  |  |  |  |  |  |  |  |  |  |  |
| *rps2* |  |  |  |  |  |  |  |  |  |  |  |  |  |  |  |  |  |  |  |  |  |  |  |  |  |  |  |  |  |  |  |  |  |  |  |  |  |  |  |  |  |  |  |  |  |  |  |
| *rps3* |  |  |  |  |  |  |  |  |  |  |  |  |  |  |  |  |  |  |  |  |  |  |  |  |  |  |  |  |  |  |  |  |  |  |  |  |  |  |  |  |  |  |  |  |  | N |  |
| *rps7* |  |  |  |  |  |  |  |  |  |  |  |  |  |  |  |  |  |  |  |  |  |  |  |  |  |  |  |  |  |  |  |  |  |  |  |  |  |  |  |  |  |  |  |  |  |  |  |
| *rps8* |  |  |  |  |  |  |  |  |  |  |  |  |  |  |  |  |  |  |  |  |  |  |  |  |  |  |  |  |  |  |  |  |  |  |  |  |  |  |  |  |  |  |  |  |  |  |  |
| *rps9* |  |  |  |  |  |  |  |  |  |  |  |  |  |  |  |  |  |  |  |  |  |  |  |  |  |  |  |  |  |  |  |  |  |  |  |  |  |  |  |  |  |  |  |  |  |  |  |
| *rps11* |  |  |  |  |  |  |  |  |  |  |  |  |  |  |  |  |  |  |  |  |  |  |  |  |  |  |  |  |  |  |  |  |  |  |  |  |  |  |  |  |  |  |  |  |  | N |  |
| *rps12* |  |  |  |  |  |  |  |  |  |  |  |  |  |  |  |  |  |  |  |  |  |  |  |  |  |  |  |  |  |  |  |  |  |  |  |  |  |  |  |  |  |  |  |  |  |  |  |
| *rps14* |  |  |  |  |  |  |  |  |  |  |  |  |  |  |  |  |  |  |  |  |  |  |  |  |  |  |  |  |  |  |  |  |  |  |  |  |  |  |  |  |  |  |  |  |  |  |  |
| *rps18* |  |  |  |  |  |  |  |  |  |  |  |  |  |  |  |  |  |  |  |  |  |  |  |  |  |  |  |  |  |  |  |  |  |  |  |  |  |  |  |  |  |  |  |  |  |  |  |
| *tufA* |  |  |  |  |  |  |  |  |  |  |  |  |  |  |  |  |  |  |  |  |  |  |  |  |  |  |  |  |  |  |  |  |  |  |  |  |  |  |  |  |  |  |  |  |  |  |  |
| *ycf1* |  |  |  |  |  |  |  |  |  |  |  |  |  |  |  |  |  |  |  |  |  |  |  |  |  |  |  |  |  |  |  |  |  |  |  |  |  |  |  |  |  |  |  |  |  |  |  |
| *ycf3* |  |  |  |  |  |  |  |  |  |  |  |  |  |  |  |  |  |  |  |  |  |  |  |  |  |  |  |  |  |  |  |  |  |  |  |  |  |  |  |  |  |  |  |  |  | N |  |
| *ycf4* |  |  |  |  |  |  |  |  |  |  |  |  |  |  |  |  |  |  |  |  |  |  |  |  |  |  |  |  |  |  |  |  |  |  |  |  |  |  |  |  |  |  |  |  |  | N |  |
| *ycf12* |  |  |  |  |  |  |  |  |  |  |  |  |  |  |  |  |  |  |  |  |  |  |  |  |  |  |  |  |  |  |  |  |  |  |  |  |  |  |  |  |  |  |  |  |  | N |  |
